# Subtype-specific expression of *MELK* is partly due to copy number alterations in breast cancer

**DOI:** 10.1371/journal.pone.0268693

**Published:** 2022-06-24

**Authors:** Ashley A. Hardeman, Yoo Jane Han, Tatyana A. Grushko, Jeffrey Mueller, Maria J. Gomez, Yonglan Zheng, Olufunmilayo I. Olopade

**Affiliations:** 1 Department of Medicine, University of Chicago, Chicago, IL, United States of America; 2 Abbott Molecular Inc, Des Plaines, IL, United States of America; 3 Department of Pathology, University of Chicago, Chicago, IL, United States of America; University of Wisconsin-Madison, UNITED STATES

## Abstract

Maternal embryonic leucine-zipper kinase (MELK) regulates cell cycle progression and is highly expressed in many cancers. The molecular mechanism of MELK dysregulation has not been determined in aggressive forms of breast cancer, such as triple negative breast cancer (TNBC). To evaluate molecular markers of *MELK* aberrations in aggressive breast cancer, we assessed *MELK* gene amplification and expression in breast tumors. *MELK* mRNA expression is highly up-regulated in basal-like breast cancer (BLBC), the major molecular subtype of TNBC, compared to luminal or other subtypes of breast tumors. *MELK* copy number (CN) gains are significantly associated with BLBC, whereas no significant association of CpG site methylation or histone modifications with breast cancer subtypes was observed. Accordingly, the CN gains appear to contribute to an increase in *MELK* expression, with a significant correlation between mRNA expression and CN in breast tumors and cell lines. Furthermore, immunohistochemistry (IHC) assays revealed that both nuclear and cytoplasmic staining scores of MELK were significantly higher in invasive ductal carcinoma (IDC) tumors compared to ductal carcinoma *in situ* (DCIS) and normal breast tissues. Our data showed that upregulation of MELK in BLBC may be in part driven by CN gains, rather than epigenetic modifications, indicating a potential for overexpression and CN gains of *MELK* to be developed as a diagnostic and prognostic marker to identify patients who have more aggressive breast cancer.

## Introduction

MELK, an atypical member of the AMPK family of serine/threonine kinases [1, 2], is involved in a variety of cellular processes including apoptosis [3], cell cycle regulation, DNA repair [4], splicing regulation [5] and hematopoiesis [6, 7]. During early development of human body, MELK is expressed by various progenitor cells, and highly expressed in the thymus, testes, spleen and certain hematopoietic progenitors of adult tissues [2, 7]. Interestingly, *MELK* is overexpressed with high proliferation index in many cancers including breast, ovarian, brain, colorectal, gastric, and blood cancers [8, 9]. As MELK expression has been suggested to have a positive correlation to histologic grade in human astrocytes and breast tumors [10], it may represent a novel prognostic marker to identify patients who have more aggressive breast cancer. However, *MELK* amplification or expression has not been evaluated as a prognostic marker to identify patients with aggressive breast cancer such as triple negative breast cancer (TNBC).

TNBC is clinically defined by tumor receptor status based on immunohistochemistry (IHC) and fluorescence *in situ* hybridization (FISH). TNBC lacks estrogen receptor (ER), progesterone receptor (PR), and epidermal growth factor receptor-2 (HER2) amplification [11]. TNBC makes up 15%-20% of all breast cancer cases, and has a relatively high rate among younger women, women of African descent, and women with *BRCA*1 mutations [12–14]. The majority of triple-negative tumors fall under the basal-like breast cancer (BLBC) molecular subtype; about 75% of TNBCs are classified as basal-like based on gene expression profiling, while the other 25% cluster with other mRNA subtypes (luminal A, luminal B, HER2-enriched or normal breast-like). Likewise, approximately 80% of BLBCs are negative for ER, PR and HER2. TNBC and BLBC are challenging to treat because of their heterogeneity and paucity of defined molecular targets.

Though patients with TNBC/BLBC have a higher response rate to neoadjuvant chemotherapy than patients with receptor-positive breast cancer, those who do not achieve pathologic complete response tend to relapse and develop distant metastatic disease. Additionally, triple-negative tumors often present with higher grades at diagnosis and display aggressive clinical behavior [11, 12]. As a result, TNBC/BLBC is associated with poor prognosis, recurrence, and shorter survival [14]. Thus, further studies are needed to identify new molecular biomarkers to help inform appropriate treatment strategies and prognoses for this subtype of breast cancer.

Understanding *MELK* aberrations, genetic mechanism(s) of *MELK* overexpression, as well as *MELK* status in breast cancer tissues could identify patients with aggressive TNBC/BLBC with poor prognosis. In this study, we examined whether gene amplification is a mechanism that may cause *MELK* overexpression in BLBC. We evaluated copy number alterations (CNA), DNA methylation, histone modifications, and *MELK* expression using publicly available databases, as well as conducting FISH, RNA-Seq, qRT-PCR, and IHC assays in breast cancer cell lines and tumors tissues. Our data showed that *MELK* copy number (CN) gains are associated with BLBC, indicating a potential role of CN gains and *MELK* overexpression as prognostic markers for patients with aggressive breast cancer.

## Results

### *MELK* is highly expressed in Basal-like breast tumors across ethnicities

**Fig 1:**
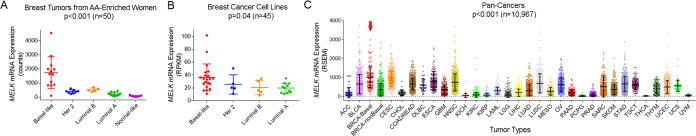
Increased expression of *MELK* mRNA in Basal-like breast cancer. (A) Subtype-specific expression of *MELK* mRNA was identified using RiboZero RNA-sequencing in breast tumors (n = 50) from women of diverse ethnicities including 66% of African Americans (AA). (B). *MELK* mRNA expression was determined in 45 breast cancer cell lines using the CCLE dataset. (C) *MELK*1 expression was analyzed using the Pan-Cancer RNA-Seq dataset. The arrow indicates *MELK* expression from basal-like breast tumors (BRCA-Basal) compared to non-Basal subtypes of breast cancer (BRCA-non-Basal) and other cancers. ACC, Adrenocortical Carcinoma; BLCA, Bladder Urothelial Carcinoma; BRCA, Breast Invasive Carcinoma; CESC, Cervical Squamous Cell Carcinoma and Endocervical Adenocarcinoma; CHOL, Cholangiocarcinoma; COADREAD, Colon Adenocarcinoma & Rectum Adenocarcinoma; DLBC, Lymphoid Neoplasm Diffuse Large B-cell Lymphoma; ESCA, Esophageal Carcinoma; GBM, Glioblastoma Multiforme; HNSC, Head and Neck Squamous Cell Carcinoma; KICH, Kidney Chromophobe; KIRC, Kidney Renal Clear Cell Carcinoma; KIRP, Kidney Renal Papillary Cell Carcinoma; LAML, Acute Myeloid Leukemia; LGG, Brain Lower Grade Glioma; LIHC, Liver Hepatocellular Carcinoma; LUAD, Lung Adenocarcinoma; LUSC, Lung Squamous Cell Carcinoma; MESO, Mesothelioma; OV, Ovarian Serous Cystadenocarcinoma; PAAD, Pancreatic Adenocarcinoma, PCPG, Pheochromocytoma and Paraganglioma; PRAD, Prostate Adenocarcinoma; SARC, Sarcoma; SKCM, Skin Cutaneous Melanoma; STAD, Stomach Adenocarcinoma; TGCT, Testicular Germ Cell Tumors; THCA, Thyroid Carcinoma; THYM, Thymoma; UCEC, Uterine Corpus Endometrial Carcinoma; UCS, Uterine Carcinosarcoma; UVM, Uveal Melanoma.

We first quantified expression of *MELK* mRNA in breast tumors, the majority of which were from women of African ancestry. Although upregulation of *MELK* in breast tumors has been reported in women of European descent [10, 15], it has not been evaluated in other ethnicities. Because TNBC/BLBC has a relatively higher prevalence among African American (AA) women, we oversampled AA women from the South Side of Chicago. We conducted RNA sequencing on fifty breast tumors from diverse patients, including 66% African Americans [16]. A subtype-specific level of *MELK* expression was observed in the AA-enriched samples, with the highest expression in BLBC subtype compared to other tumor subtypes (p < 0.001) (Fig 1A).

We next determined *MELK* expression in breast cancer cell lines using Cancer Cell Line Encyclopedia (CCLE) databases, which showed a significant increase in *MELK* mRNA in BLBC cell lines compared to other subtype cells (p = 0.04) (Fig 1B). To compare *MELK* expression across various cancers, we utilized RNA-Seq data in pan-cancer samples of The Cancer Genome Atlas (TCGA) and analyzed *MELK* expression in 33 types of human cancers (n = 10,967). The highest *MELK* expression levels were observed in BLBC tumors (BRCA-Basal) compared to non-BLBC tumors (BRCA-non-Basal) as well as to all other tumors (Fig 1C). Cervical squamous cell carcinoma (CESC) showed the second highest levels of *MELK* expression among all other tumors compared. Collectively, the data from TCGA, CCLE, and AA-enriched samples showed that *MELK* is highly expressed in BLBC tumors compared with all types of cancers.

### Subtype-specific expression of *MELK* is not due to epigenetic changes

**Fig 2:**
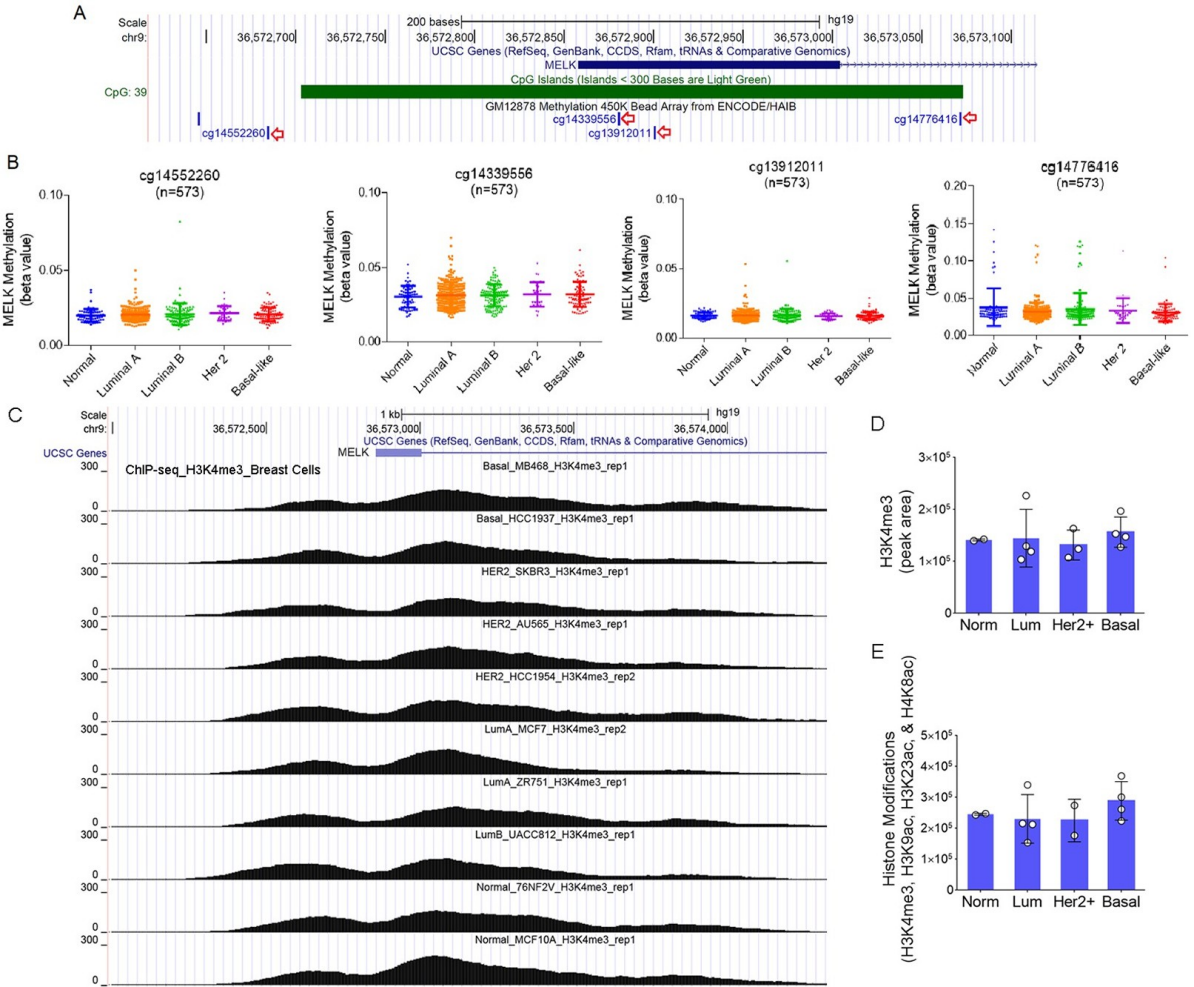
Epigenetic regulation of the *MELK* promoter in breast tumors. (A) The UCSC genome browser shows the location of CpG islands in the *MELK* promoter at chromosome 9p13.2. The four CpG dinucleotides located closest to the transcription start site were selected for analysis and are indicated by red arrows. (B) Analysis of TCGA HumanMethylation450 Array data showed no difference in CpG site methylation among breast cancer subtypes. Beta values (0 to 1) are relative values increasing from hypomethylation to hypermethylation. (C) ChIP-seq data for H3K4me3 in breast cancer cells were visualized through the UCSC genome browser (chr9:36,571,990–36,574,891). Each peak represents the level of H3K4me3 modification in each cell line. (D and E). The levels of H3K4me3 (C) and other histone modifications (H3K4me3, H3K9ac, H3K23ac and H4K8ac combined) (D) were calculated as a total peak area (Y-axis, details in the methods) and compared by molecular subtype. No significant differences in histone modifications were observed among breast cancer subtypes.

Upregulation of *MELK* mRNA in BLBC can be influenced by epigenetic modifications, CNA, or other regulatory factors. We thus examined whether epigenetic regulation of the *MELK* promoter contributes to the subtype-specific expression of *MELK* by analyzing methylation levels of CpG dinucleotides in the promoter using the TCGA Human Methylation450 Array data. The UCSC genome browser view showed the CpG site locations in the promoter region on chromosome 9p13.2, including the four sites analyzed (cg14552260, cg14339556, cg13912011) (Fig 2A). Although the promoter is generally hypomethylated and activated in breast tumors, there was no significant difference in CpG site methylation of the promoter among BLBC and other subtypes (Fig 2B).

We next determined the levels of histone modifications in the *MELK* promoter using ChIP-seq data from 13 different human breast cell lines [17]. No significant difference in H3K4me3 modification, the major histone modification in the promoter, was observed among different molecular subtypes of breast cancers (Fig 2C and 2D). When we combined all other histone modifications in the promoter (H3K4me3, H3K9ac, H3K23ac, and H4K8ac), we did not observe any significant differences among breast cancer subtypes either (Fig 2E). Collectively, the data indicate that increased expression of *MELK* in BLBC is not due to epigenetic changes of DNA methylation or histone modifications in the promoter.

### *MELK* expression is modestly correlated with copy number in breast cancer

**Fig 3:**
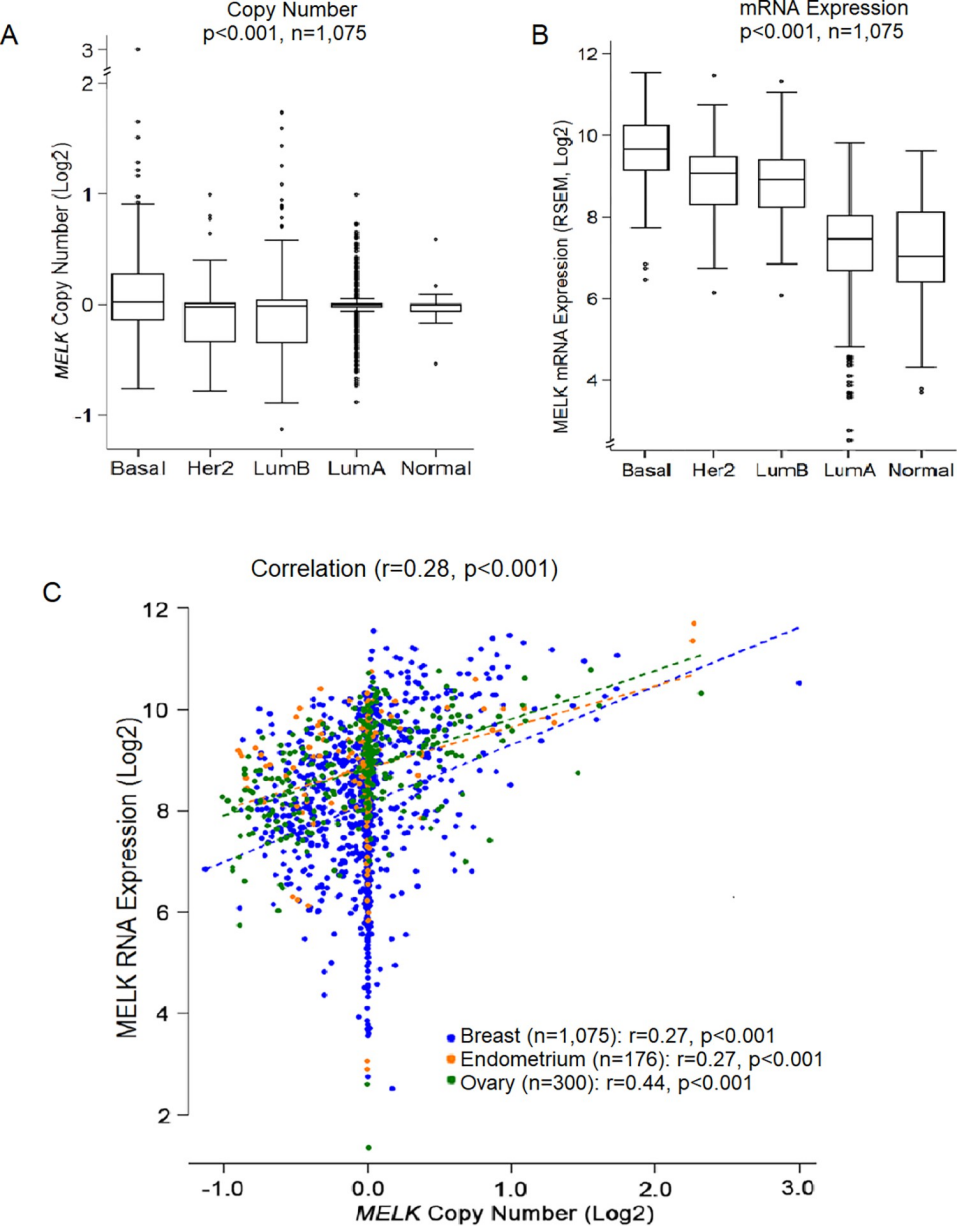
Correlation between *MELK* gene copies and mRNA expression in breast tumors. (A) Analysis of TCGA CNA dataset (Affymetrix SNP Array 6.0) showed that alterations in *MELK* DNA CN is significantly correlated with breast cancer subtypes (n = 1,075, p<0.001), with the highest CN gains in basal-like breast cancer compared to other subtypes of breast cancer (basal *vs* other subtypes, p<0.001). (B) *MELK* mRNA expression is strongly correlated with breast cancer subtypes (n = 1,075, p<0.001), with the highest expression in basal-like tumors (basal *vs* other subtypes, p<0.001). (C) *MELK* mRNA expression showed a significant correlation with *MELK* DNA CN in primary breast, endometrial, and ovarian cancers from TCGA datasets (n = 1,551, p<0.001). The X-axis represents log2 CN and the Y-axis represents log2 RNA transcripts. *P*-values were calculated using Pearson correlation analysis.

We tested whether subtype-specific expression of *MELK* is due to CNA of the *MELK* gene. TCGA breast cancer dataset demonstrated a moderate and significant increase in *MELK* gene copies in the BLBC subtype (p<0.001) (Fig 3A). *MELK* transcript levels are also much higher in the basal subtype (Fig 3B). Importantly, when we conducted an assessment of *MELK* CNA and gene expression data from 1551 TCGA woman’s cancers (1075 breast, 176 endometrial, and 300 ovarian), we found a moderate and significant correlation between CN and expression of *MELK* (r = 0.28, p<0.001) (Fig 3C).

**Fig 4:**
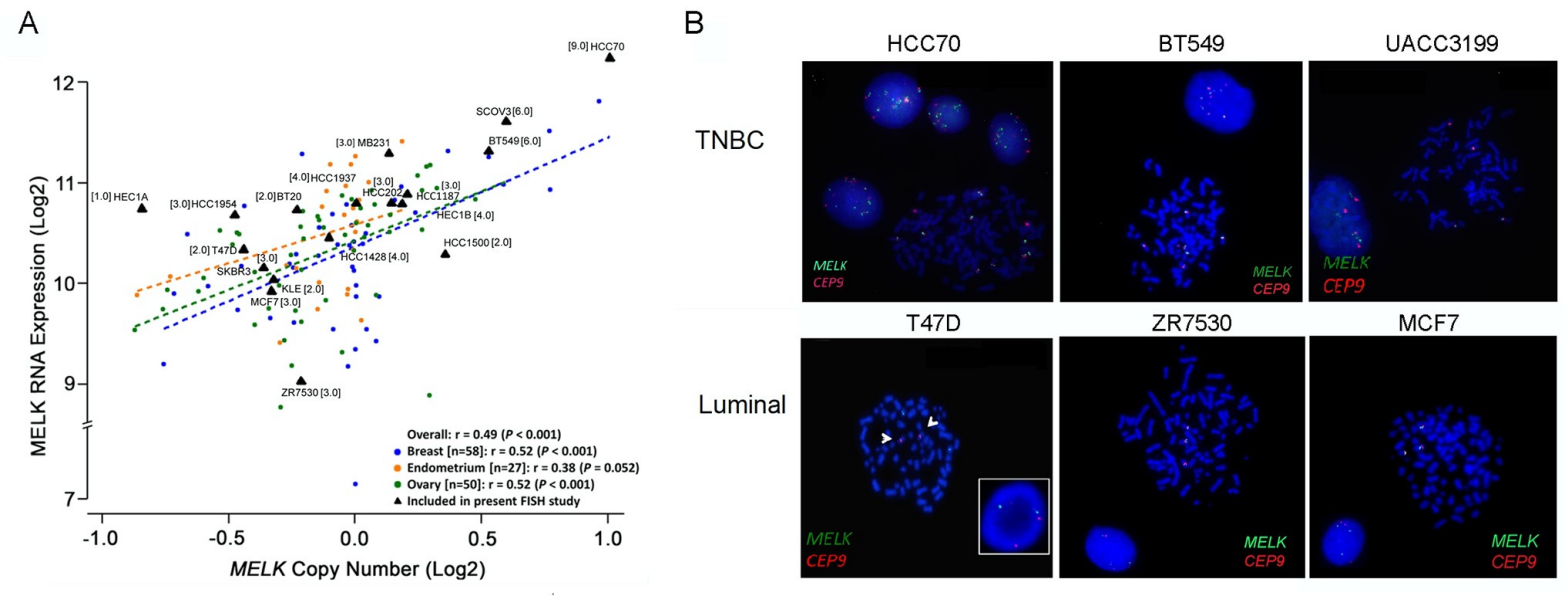
Correlation of *MELK* mRNA expression with copy numbers in breast cancer cell lines. (A) *MELK* RNA expression moderately correlates with *MELK* DNA CN in breast (r = 0.52, p<0.001), endometrial (r = 0.38, p<0.001), and ovarian cancer (r = 0.52, p<0.001) cell lines from the CCLE cohort (n = 135). The X-axis represents CN and the Y-axis represents RNA transcripts. *P*-values were calculated using the Pearson correlation test. Labeled black triangles mark 18 cell lines analyzed by *MELK*/*CEP9* FISH in present study. Brackets are the absolute mean *MELK* copies/cell. (B) FISH images of six representative cell lines (three TNBC and three luminal cell lines) with *MELK* gain and loss are given for comparison. *MELK* is localized by the green fluorescent signal and the *CEP9* is localized by the red fluorescent signal. The cells were counterstained with DAPI (blue). The arrowhead indicates a structural alteration. Detailed FISH results are summarized in Table 1.

To further explore the relationship between *MELK* expression with CN, we analyzed *MELK* CNA in 135 cell lines of female cancers (58 breast, 27 endometrial, and 50 ovarian) using the CCLE single nucleotide polymorphism (SNP) array dataset. Analyses of *MELK* CNA and gene expression data revealed that *MELK* expression was also significantly correlated with CN across breast cancer cell lines (r = 0.52, *p <* 0.001) as well as across all cell lines of female cancers (r = 0.49; *p <* 0.001) (Fig 4A). The data suggest that CN changes of the gene may regulate *MELK* expression in breast cancer.

We then evaluated *MELK* CNA by FISH in breast cancer cell lines. When we determined the probe hybridization efficiency in the normal lymphocyte cell line GM14667, we observed a normal pattern of two copies of each signal with a *MELK*:*CEP9* (centromere enumeration probe for chromosome 9) ratio of 1.0 (S1 Fig in S1 File). However, breast cancer cells displayed frequent abnormal signal patterns including low to high chromosome polysomy (Table 1 and S2 Fig in S1 File). In particular, many basal-like cell lines were highly polysomic for chromosome 9 and *MELK*, compared to luminal subtype cells (Fig 4B and S3 Fig in S1 File). CN gains in two BLBC cell lines (HCC70 and BT549) were observed, which were also identified by CCLE SNP arrays as amplification and low-level gain of *MELK*, respectively. In contrast, the majority of the luminal cell lines had low levels of polysomy. Collectively, the correlation of *MELK* gene copies with mRNA in breast tumors and cancer cell lines suggest that subtype-specific expression of *MELK* may be partly due to CNA in breast cancer.

**Table 1:** *MELK* gene copies and RNA expression in breast cancer cell lines.

Organ of origin and molecular subtype	Cell line	*MELK* FISH					*MELK* mRNA		
							(qRT-PCR)		
		*MELK*/cell^1^	*CEP9*/cell^1^	*MELK/CEP9*	Major clone *MELK*:*CEP9* (%)^3^	Interpretation^4^	RQ^5^	SD	Interpretation^6^
				Ratio^*2*^					
Breast cancer	HCC70	8.9	10.6	0.8	9:10–15 (55%)	High unbalanced polysomy	381.55	25.73	High
	BT549	6.4	7.7	0.8	6:8 (19%), 8:9 (15%)	High unbalanced polysomy	N/D	N/D	N/D
	UACC3199	4.0	4.0	1.0	4:4 (65%)	High balanced polysomy	86.04	8.27	High
Triple-negative	HCC1187	3.0	2.9	1.0	3:3 (90%)	Low balanced polysomy	57.38	4.87	High
/Basal	MDAMB231	3.2	3.1	1.0	3:3 (85%)	Low balanced polysomy	44.18	3.87	Moderate
(ER-/PR-/HER2-)	HCC1954	3.1	3.8	0.8	3:4 (80%)	Low unbalanced polysomy	41.29	2.06	Moderate
	BT20	2.0	3.0	0.7	2:3 (82%)	SA, unbalanced disomy	38.36	3.12	Moderate
	HCC1937	3.8	3.8	1.0	4:4 (63%)	High balanced polysomy	34.51	9.63	Moderate
	HCC1500	2.0	2.1	1.0	2:2 (80%)	Disomy balanced	29.11	1.86	Moderate
Breast Cancer	HCC1428	3.8	3.8	1.0	4:4 (73%)	High balanced polysomy	50.78	2.86	High
Luminal A	T47D	2.0	1.2	1.7	2:1 (77%)	SA, *CEP9* hemizygous deletion	27.43	1.71	Moderate
(ER+/PR+/HER2-)	MCF7	3.0	3.0	1.0	3:3 (80%)	Low balanced polysomy	12.90	1.02	Low
Breast cancer	ZR7530	3.0	3.0	1.0	3:3 (88%)	Low balanced polysomy	19.05	0.75	Low
Luminal B									
(ER+/PR+/HER2+)									
Breast cancer	HCC2185	29.1	5.3	5.5	-	High ampl; *CEP9* high polysomy	265.44	16.2	High
HER2+	HCC202	3.0	5.1	0.6	3:5 (58%)	Unbalanced polysomy	28.07	1.10	Moderate
ER-/PR-/HER2+)	SKBR3	3.1	3.0	1.1	3:3 (80%)	Low balanced polysomy	5.09	0.43	Low
Normal breast	HMEC	N/D	N/D	N/D	N/D	qRT-PCR Control	1.00	0.04	Baseline
Normal lymphoblasts	GM14667	2.0	2.0	1.0	2:2 (90%)	FISH Control	N/D	N/D	N/D

^1^mean copies of *MELK* or chromosome 9 centromere enumeration probe (*CEP9*) per cell

^2^ mean gene to chromosome ratio per cell

^3^ most representative clone of cells with given copies of *MELK* and *CEP9* per cell

^4^FISH interpretation: Amplification (Ampl), *MELK to CEP9* ratio ≥ 2.0; polysomy, *MELK* and *CEP9* copy number ≥3; balanced polysomy, equal copy number gain; unbalanced polysomy, unequal copy number gain; low polysomy, copy number >2 and ≤ 3; high polysomy, copy number ≥4; SA, structural alterations, rearrangements of chromosome 9 resulting in *MELK* loss, duplication or translocation

^**5**^RQ: Relative Quantification, mRNA value relative to HMEC control (2-ddCt method)

^6^*qRT-PCR* interpretation: High (RQ ≥ 50), moderate (20 ≤ RQ <50) or low (1 < RQ < 20) expression

N/D, no data

### *MELK* protein expression is increased in invasive ductal carcinoma (IDC) tumors

To test the possibility for *MELK* to be developed as breast cancer prognostic marker, we conducted IHC in 87 human breast tissue samples: IDC (n = 39), ductal carcinoma *in situ* (DCIS) (n = 10), metastases to lymph nodes (n = 5), and benign (n = 33). Benign colon tissue served as a positive control (S4 Fig in S1 File).

**Fig 5:**
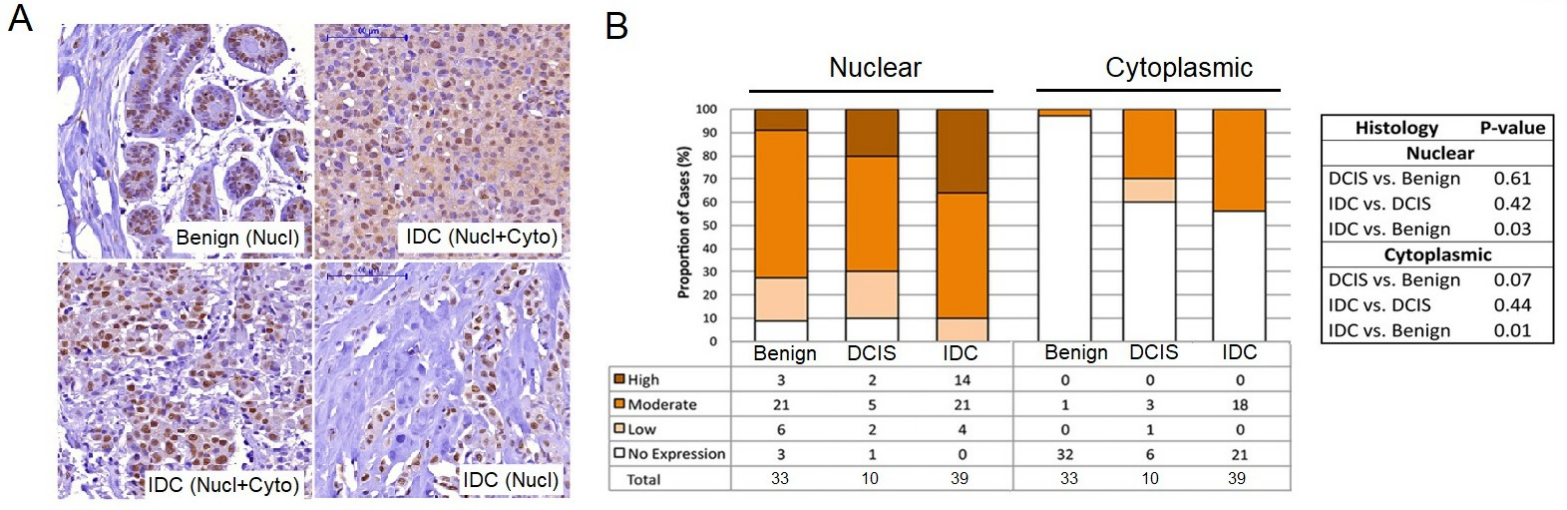
Increased expression of *MELK* proteins in invasive ductal carcinoma (IDC) compared to benign tissues. (A) Representative images from immunohistochemistry (IHC) analyses of *MELK* protein expression in benign epithelia and IDC tissues. *MELK* expression was interpreted using the ImmunoReactive Scoring system (IRS), on a scale of 0–12, as no/low (scores 0–3, negative) and moderate/high (scores 4–12, positive). Benign tissue with only nuclear (Nucl) expression (moderate IRS score 8) in 90% of cells is shown. IDC from a luminal A tumor presented with moderate *MELK* expression in both nuclei (score 8) and cytoplasm (score 4) (Nucl+Cyto). Examples of two *MELK*-positive IDCs of the same histological grade 3 and stage 2A, showing the presence and absence of cytoplasmic staining, respectively. (B) Nuclear and cytoplasmic expressions of *MELK* proteins are quantified in benign, DCIS and IDC tissues, which was significantly higher in IDC compared to benign tissues (p = 0.03 and 0.01, respectively). Bars represent the proportion of cases with the given resignation. *P*-values were calculated using either Kendall’s tau-b for group comparison or cumulative link mixed models for pairwise comparisons and adjusted by Holm’s method.

As the *MELK* protein localizes to both nucleus and cytoplasm of breast epithelial tissues, we assessed each cellular location independently or in combination (Fig 5 and S1 Table in S1 File). There was significantly increased nuclear and cytoplasmic MELK staining in advanced tumor compared with benign tissues. The percentages of MELK-expressing nuclei (mean ± SD) were 64.85 ± 32.89 in benign, 69.0± 41.34 in DCIS, and 87.18 ± 24.6 in IDC (p *=* 0.005) (Fig 5B). The cytoplasmic intensity was lower than nuclear staining and was detected only in tissues with nuclear staining. Positive cytoplasmic staining for MELK ranged from 3% (1/33) of benign, to 40% (4/10) of DCIS cases, and to 44% (17/39) of IDC cases (p<0.001).

Using the ImmunoReactive Scoring (IRS) system (intensity x % positive cells score), MELK staining was classified as no expression, low, moderate or high expression. By pairwise comparisons, both nuclear and cytoplasmic IRS scores were found to be positively associated with IDC, with significantly increased MELK expression in IDC compared with benign tissues (nuclear, p *=* 0.03; cytoplasmic, p *=* 0.01), while MELK expression in DCIS was intermediate. The data demonstrated higher expression of MELK in patients with IDC tumors compared to those with benign tissues or DCIS tumors.

## Discussion

Although *MELK* has been shown to be significantly up-regulated in breast tumors and to be involved in cell cycle regulation and apoptosis, little is known about the genetic and regulatory factors contributing to the altered expression of *MELK* in BLBC. In this study, we have shown that *MELK* expression is highly increased in BLBC compared to other subtypes of breast cancer from AA-enriched women (Fig 1A). Pan-cancer evaluation of *MELK* expression also revealed the greatest expression in BLBC tumors (BRCA-Basal) compared to all other tumors (Fig 1C). The subtype-specific expression of *MELK* in breast tumors is not associated with epigenetic modifications of the CpG islands or histones of the promoter (Fig 2). In contrast, CN gains of *MELK* are associated with BLBC, showing significant correlation between mRNA expression and CN in breast tumors and cell lines (Figs 3 and 4). Moreover, both nuclear and cytoplasmic expression of *MELK* proteins were significantly higher in IDC tumors compared to DCIS and normal breast tissues (Fig 5). Our data suggest that overexpression and CN gains of *MELK* can be developed as a diagnostic and prognostic marker to identify patients who have more aggressive breast cancer.

It is notable that the *MELK* gene is located within the pericentromeric region of chromosome 9 (9p13.2) that harbors several tumor-related genes. Certain CNA such as amplifications in this region have been associated with cancer development [18] and resistance to chemotherapy [19]. However, data on the involvement of *MELK* alterations of this region have been limited. In this study, we showed that the gain of *MELK* gene copies was a common alteration in cancers of the breast, endometrium, and ovary (4.7%-50%). Overall, the correlation of gene copies with mRNA (r = 0.28, p*<*0.001) suggests that *MELK* gene gains through chromosome polysomy might contribute to elevated gene and protein expressions in a subset of cases, although the *MELK* locus is not a primary target for amplifications in breast and other female cancers.

It appears that in addition to CNA, other factors may regulate the expression of *MELK* during tumorigenesis. Previous studies have shown that a high level of *MELK* overexpression in BLBC is partly dependent on FOXM1, a master mitotic transcription factor that is found to be highly overexpressed in BLBC. *MELK* interacts with FOXM1 in the nucleus, phosphorylates and activates it, forming the transcriptional complex *MELK*-FOXM1-TOPK [20, 21]. This complex regulates expression of cell cycle genes, DNA replication, DNA damage responses, and cell proliferation. Therefore, it is possible that both CN gains and FOXM1 upregulation contribute to overexpression of *MELK* in BLBC, warranting further investigations. The localization of *MELK* protein seems dynamic and regulated in a cell-cycle dependentmanner [22]. We detected MELK protein both in nuclei and cytoplasm of breast tumors using previously validated anti-MELK antibody [23, 24], while human protein atlas data showed that MELK protein was mainly detected in cytoplasm, or both in nuclei and cytoplasm of breast tumors. The data suggest that MELK kinase has broad substrate specificity and is involved in multiple cellular processes. Many of these process require nuclear localization, particularly in cancer cells, including an interaction with transcription factor FOXM1 [20]. We found that *MELK* protein in either nuclear, or cytoplasmic, or combined nuclear+/cytoplasmic+ compartments was significantly higher in IDC compared with benign tissues, suggesting the important role of *MELK* in the occurrence and progression of breast cancer.

Collectively, our findings confirm that *MELK* expression is significantly upregulated in aggressive breast cancer and is associated with the gain of gene copies as the main alteration. The data suggest the significant role of *MELK* in aggressive breast cancer and supporting further investigation of the *MELK* mRNA/protein level as a biomarker for identifying candidates who may benefit from *MELK*-targeted therapy.

## Materials and methods

### Cell lines

Breast cancer cell lines BT20, BT549, HCC70, ZR7530, HCC1187, HCC1937, HCC1500, HCC1954, HCC1428, HCC202, MCF7, MDAMB231, SKBR3 and T47D, ovarian cancer SK-OV-3 and endometrial cancer KLE cells were purchased from the American Type Culture Collection (ATCC, Manassas, VA). Breast cancer cell lines HCC2185 and UACC3199 were purchased from the University of Texas Southwestern Medical Center (UTSM, Dallas, TX) and the University of Arizona Cancer Center (UACC, Tucson, AZ), respectively. Endometrial cancer cells HEC1A and HEC1B were provided by Dr. Ernest Lengyel (University of Chicago Medical Center, Chicago IL). Human mammary epithelial cells (HMECs) were purchased from Lonza, Inc. The lymphoblastoid cell line GM14667 was established from a normal individual and was barcoded in our research laboratory. HMEC and GM14667 were used as experimental controls. All cell lines tested negative for mycoplasma contamination and were validated for species and unique DNA profile using short tandem repeat (STR) analysis by the provider or in our laboratory. Cell lines were cultured in a humidified atmosphere of 5% CO_2_ at 37°C according to the providers’ recommendations, in appropriate media containing 10% FBS, 100uL penicillin G, and 0.1mg/mL streptomycin (1% penicillin/streptomycin) (Sigma-Aldrich).

### Patient material

This study was conducted under research protocols approved by the University of Chicago Institutional Review Board (13304B and 16352A), under which all participating patients signed a written informed consent. Formalin-fixed paraffin-embedded (FFPE) breast cancer tissue samples mounted on a tissue microarray were obtained from the University of Chicago Breast Cancer Tissue Repository [25]. The histology of each tissue core in hematoxylin and eosin stained slides was verified by two pathologists independently. Eighty-six representative cores of IDC (n = 39), DCIS (n = 10), metastatic (n = 4) and benign epithelial tissues (n = 33) were analyzed. Clinical and pathological features including race, age, tumor size, histological type, tumor grade, ER, PR and HER2 receptor statuses, nodal involvement were collected.

### qRT-PCR

Total cellular RNA were extracted from cultured cells using the RNeasy mini kit (Qiagen, Montgomery, MD). The integrity of RNA was validated using the bio-analyzer at the University of Chicago Genomics Core Facility (https://fgf.uchicago.edu). For cDNA synthesis, reverse transcriptase reactions were done using the SuperScript III First Strand Synthesis System (ThermoFisher Scientific, Waltham, MA) with 1 ug of RNA. All qRT-PCR reactions were performed in quadruplicate within the 7900HT Fast Real-Time PCR System apparatus (ThermoFisher Scientific, Waltham, MA), using the TaqMan Gene Expression Master Mix or Power SYBR Green PCR Master Mix (ThermoFisher Scientific, Waltham, MA), along with *MELK* probe assays (Hs01106440_m1). The fold change in *MELK* cDNA (target gene) relative to the *18s* rRNA endogenous control determined the relative quantification value (RQ) by the ΔΔCt method. Based on the RQ values, cell lines had high (RQ ≥ 50), moderate (20 ≤ RQ <50) or low (1 < RQ < 20) *MELK* mRNA expression (Table 1). HMEC was used as a control for baseline *MELK* expression.

### FISH

Dual-color FISH assays were conducted using a *MELK*:*CEP9* probe mixture containing custom-made *MELK* DNA (BAC clone RP11-450B8) labeled with *SpectrumGreen* and the *SpectrumOrange CEP9* (Abbott Molecular, Downers Grove, IL). *CEP9* was used to distinguish true gene amplification from CN gain due to chromosome 9 polysomy. The *MELK* probe was directly labeled using the Nick Translation Kit (Abbott Molecular, Downers Grove, IL). Chromosomal mapping and hybridization efficiency for the probe mixture was verified in metaphase spreads of normal lymphoblastoid cells GM14667 (S1 Fig in S1 File). Metaphase cell preparations of cell lines were done according to routine protocols [26]. The pretreatment of FFPE tissue sections and all hybridization procedures and post-hybridization washes were done as described by Abbott Molecular. Mean copies of *MELK* and *CEP9* per cell were scored and CN ratios of *MELK* to *CEP9* were calculated. A ratio of *MELK to CEP9* ≥ 2.0 was a cut off point for *MELK* amplification. The gain in gene signals to ≥ 3 due to polysomy for chromosome 9 was classified as gene polysomy. Balanced (equal CN gain of both signals) and unbalanced (unequal CN gain) polysomy, low polysomy (CN = 3) and high polysomy (CN ≥ 4) were recorded. Rearrangements of chromosome 9 resulting in *MELK* loss, duplication or translocation were marked as structural alterations.

### IHC

*MELK* protein expression was identified using the same primary mouse anti-*MELK* antibody (1:3,000) as for Western blotting. Antibody specificity and sensitivity were validated previously [23]. The IHC procedure was done at the University of Chicago IHC Core Facility, which applied the Histofine Simple Stain MAX-PO (mouse) detection system (B-Bridge International, CA). Sections of benign colon and colon cancer tissues were selected as positive controls (S2 Fig in S1 File). Isotype staining with the corresponding immunoglobulin instead of Ab was used as a negative control for antibody specificity. Staining intensity, percentage of positive cells and localization (nuclear or cytoplasmic) were recorded. Each sample was scored in a blinded fashion by two pathologists in a semi-quantitative manner. MELK expression was interpreted using the IRS system as described by Faggad and colleagues [27]. Namely, the immunoreactivity of MELK antibody was labeled as 0 (negative), 1+ (weak), 2+ (moderate) and 3+ (strong). The percentage of immunostained cells was captured at each intensity level and graded as following scores: 0 (0% staining), 1 (staining in 1–10% of tumor cells), 2 (11–50%), 3 (51–80%) and 4 (> 80%). The intensity staining multiplied on percentages of positive cells score resulted in combined score with values between 0 and 12. Scores of 0 (no expression) and 1–3 (low expression) were designated as negative, whereas scores of 4–12 were designated as positive (4–8, moderate; > 8, high) expression.

### Analysis of public databases

*MELK* CNA and mRNA expression profiles in breast, ovarian and endometrial cancer cell lines (n = 135) from the CCLE were downloaded from cBioPortal. In this dataset the DNA CNA was detected by Affymetrix SNP Array 6.0, and gene expression levels were detected by Affymetrix U133 Plus 2.0 Arrays. Queries on CNA (GISTIC2 method) and mRNA expression (RNA-seq RSEM) profiles for breast, endometrial, and ovarian cancers were accessed and analyzed using the cBioPortal (http://www.cbioportal.org) as recommended [28].

### ChIP-seq data analysis

ChIP-seq data for H3K4me3, H3K9ac, H3K23ac and H4K8ac in breast cancer cell lines were downloaded from https://www.ncbi.nlm.nih.gov/geo/query/acc.cgi?acc=GSE85158 [17] and visualized through UCSC Genome Browser custom tracks (hg19, chr9:36,571,990–36,574,891). Measurement of the total peak area in this region is the summation of a number of segment contents (distance between two chromosomal coordinates x ChIP-seq signal value) over all segments within the peak. As peaks consist of multiple bins (rectangles) in the track graph, each peak area was calculated by multiplying chromosomal ordinates (width) x signal (height) and then peak area in the region was calculated as the sum of the areas.

### Statistical analysis

For comparisons of nuclear percent of stained cells as a continuous outcome across tissue, the non-parametric Kruskal-Wallis test and the parametric analysis of variance were applied. For comparisons of percent of stained cells [continuous outcome for two types of tissues (ordinal groups)], non-parametric two-sided Wilcoxon rank sum test and parametric two-sided t-test were used. For comparison of binary and multi-level scores/ratings [nuclear and cytoplasmic intensities and IRS final score group for both nucleus and cytoplasm, (ordinal) across Normal, DCIS, and IDC histological types], the Kendall’s tau-b test correlated ordinal variables with tied ranks was used. Statistical significance was defined as p<0.05 at two sides. All analyses were performed using R version 3.3.1, Stata version 14 (StataCorp, Chicago, IL), or GraphPad Prism 6.0 (GraphPad Software, Inc., La Jolla, CA).

## Supporting information

S1 File Supporting information includes four figures (S1-S4 Figs) and one table (S1 Table). (DOCX)
