# Supplementary material for: Subtype-specific expression of MELK is partly due to copy number alterations in breast cancer
Source: PLoS One. 2022 Jun 24;17(6):e0268693. doi: 10.1371/journal.pone.0268693 (PMC9231703; doi:10.1371/journal.pone.0268693)
Supplement: S1 File — (DOCX) [file pone.0268693.s001.docx]

# A


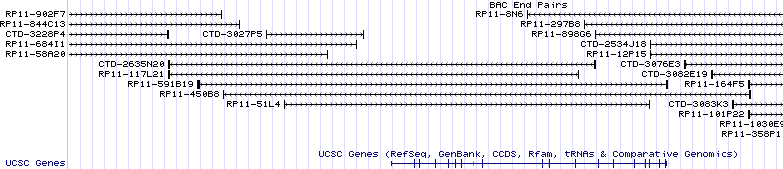

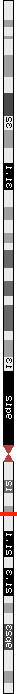

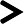


http:/genome.ucsc.edu/cgi-bin/

BAC RP11-450B8 size ≈ 200kb

9p13.2

*MELK* gene size ≈ 105kb


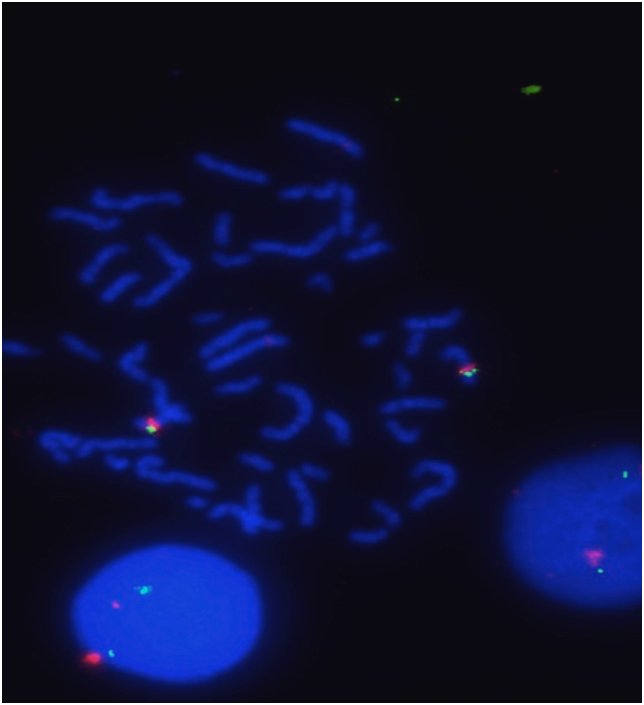


B Normal Lymphoblastoid cell line

*MELK CEP9*

Chromosome 9

**S1 Fig. *MELK* selected clone, probe validation and FISH assay.** (**A**) BAC clone RP11-450B8 (http:// genome.ucsc.edu/cgi-bin) contains the full-length of 105 kb *MELK* DNA with the optimal probe size of 200 kb. (**B**) An image of *MELK:CEP9* FISH in control normal human lymphoblastoid cell line in metaphase and interphase is presented. The *MELK* gene is localized by green fluorescent signal, *CEP9* is localized by red fluorescent signal. The *MELK* probe gave adequate bright signals. The normal pattern of two signals per cell for *MELK* and *CEP9* probes can be detected on chromosome 9 (see also Table 1). The cells were counterstained with DAPI (blue). Original magnification x630.


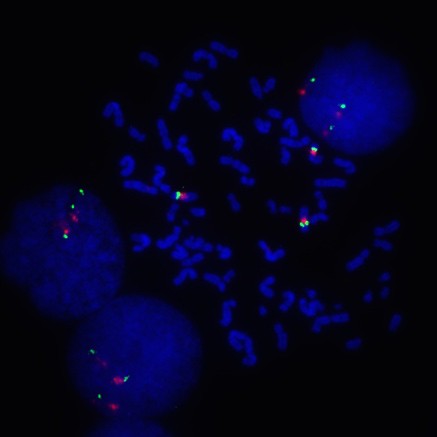

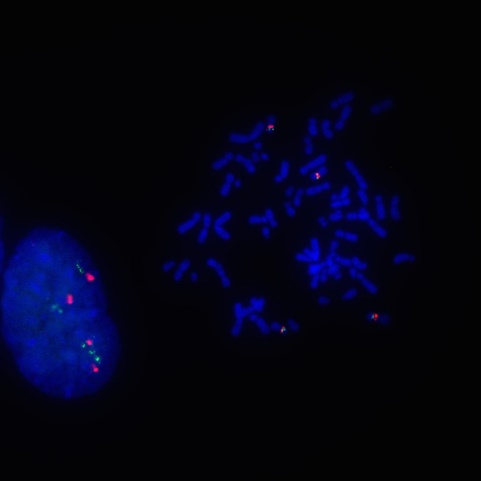

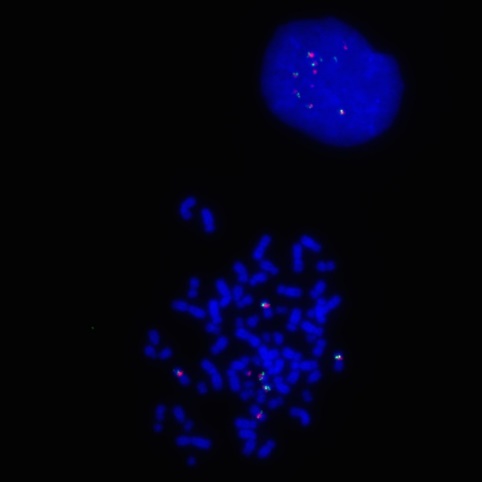

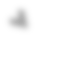

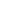


**BT549**

TNBC **UACC3199**

TNBC **HCC1187**

TNBC

*MELK CEP9*

*MELK CEP9*

*MELK CEP9*


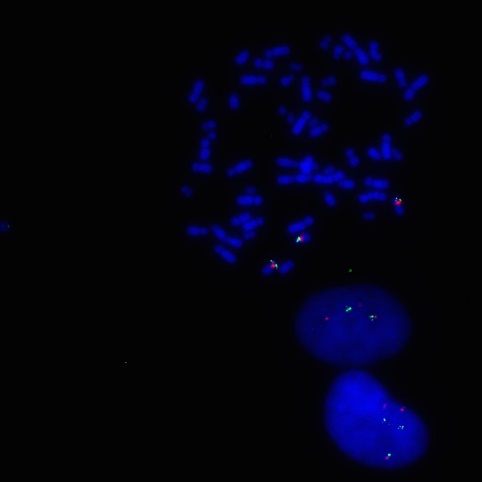


**MDAMB231**

TNBC

*MELK CEP9*


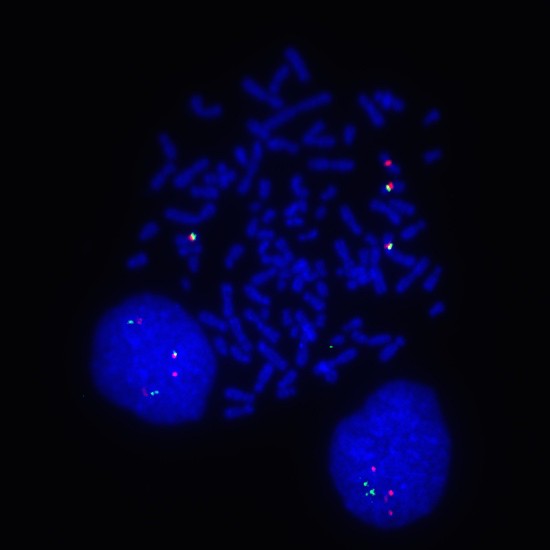

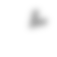

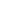


**HCC1954**

TNBC

*MELK CEP9*


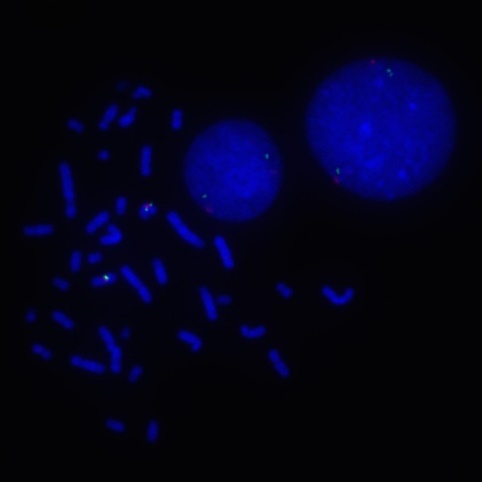


**HCC1500**

TNBC

*MELK CEP9*


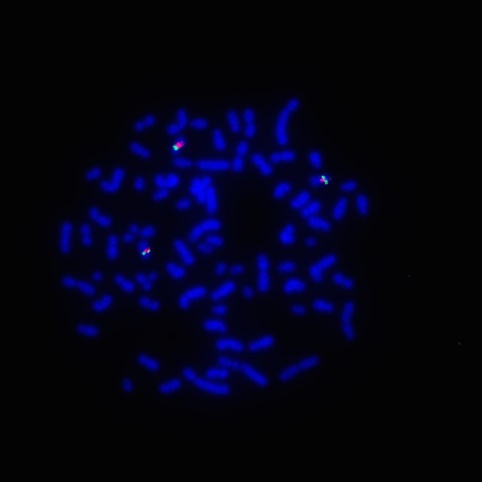

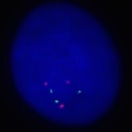

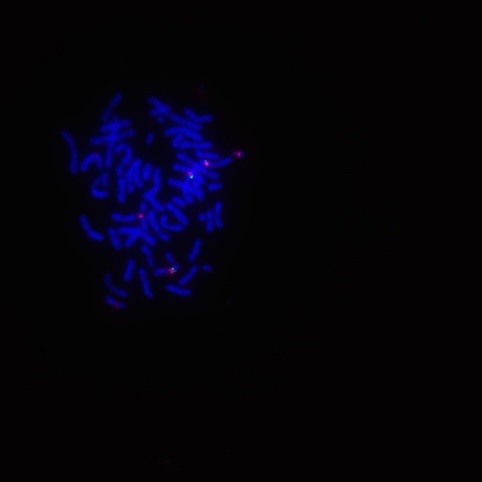

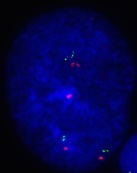

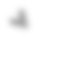

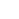

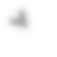

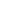


**HCC202**

HER2+ **SKBR3**

HER2+

*MELK CEP9*

*MELK CEP9*


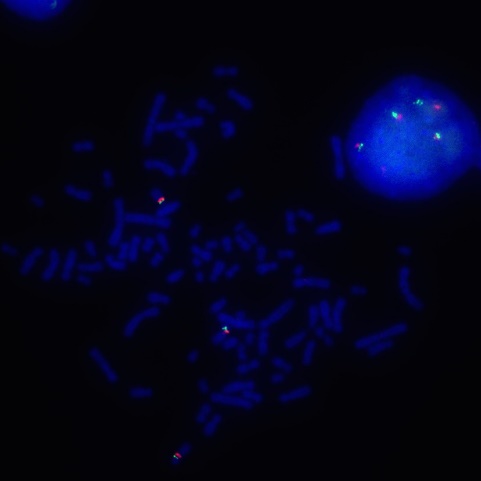


**HCC1428**

LumA

*MELK CEP9*


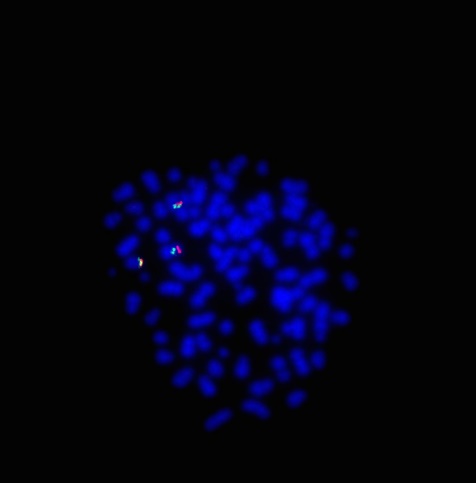


MCF7

LumA

*MELK CEP9*


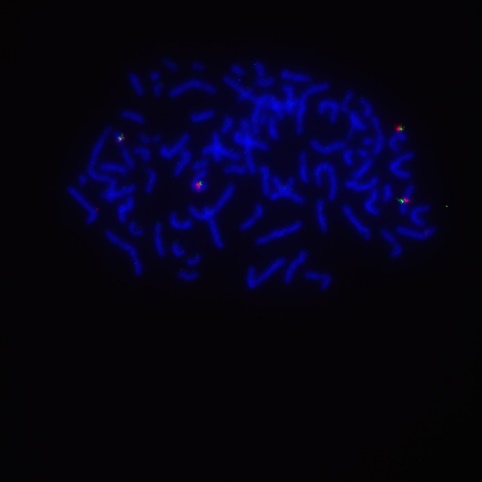

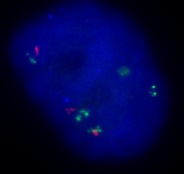

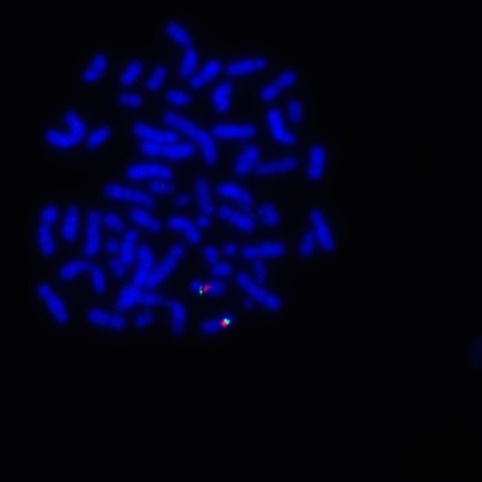

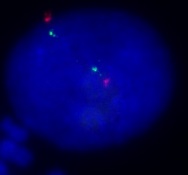


**KLE**

Endometrial **HEC1B**

Endometrial

*MELK CEP9*

*MELK CEP9*


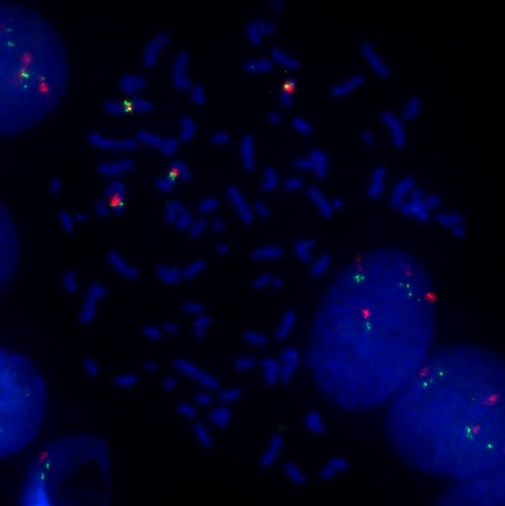

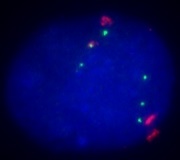

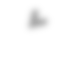

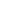


SKOV3 (Ovary)

*MELK CEP9*


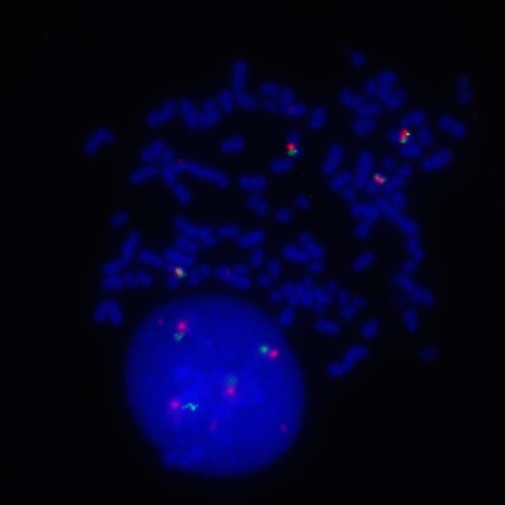


HCC1937 (TNBC)

*MELK CEP9*


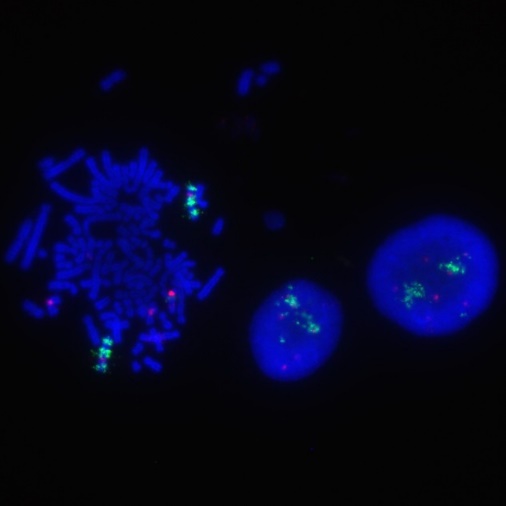

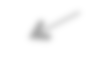

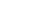

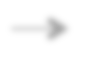


HCC2185 (HER2+)

*MELK CEP9*


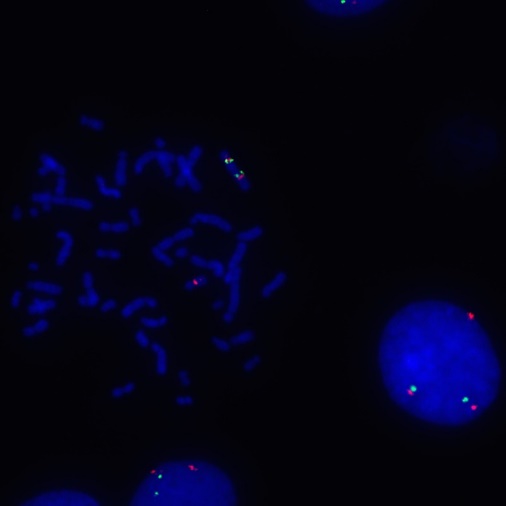

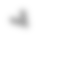

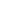

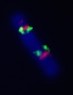

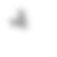

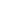


BT20 (TNBC)

*MELK CEP9*

**S2 Fig. Representative FISH images of *MELK* alterations in cancer cell lines.** The *MELK* gene is localized by the green fluorescent signal; chromosome 9 centromere enumeration probe (*CEP9)* is localized by the red fluorescent signal. The cells were counterstained with DAPI (blue). Original magnification x630. In UACC3199, HCC1428 and HEC1B the presence of two extra copies of both signals defined high balanced and unbalanced tetrasomy. BT549 cells show high unbalanced polysomy with >6 copies of each signal.

HCC1187, MDAMB231, HCC1954, SKBR3, HCC202 and MCF7 cells carried one extra copy of *MELK* and 1-3 extra copies of chromosome 9, and were classified as having balanced and unbalanced low polysomy. HCC1500 and KLE cells showed two copies of each signal (balanced disomy). HCC2185 cells showed high amplification. Multiple copies of *MELK* (13–50/nucleus) are located within intrachromosomal amplicons on two chromosomes 9; three extra copies of the red signal show high polysomy for a derivative of chromosome 9. BT20 cells carry dicentric chromosome 9 with duplicated *MELK* and one extra copy of chromosome 9 derivative (unbalanced low polysomy). HCC1937 cells show trisomy for chromosome 9 with concordant gain of *MELK* (balanced polysomy). SKOV3 ovarian cancer cells were polysomic by carrying three copies of chromosome 9 with intact *MELK* and one chromosome 9 derivative with duplicated *MELK*. Arrow indicated gene amplification and arrowhead indicated structural alteration.

**S3 Fig.** *MELK* DNA copy numbers were analyzed by FISH and compared between basal-like (n=9) and luminal subtypes (n=4) in thirteen breast cancer cell lines. FISH images were shown in Figure 4B and supplementary Figure S2.

## Positive Control Negative Control


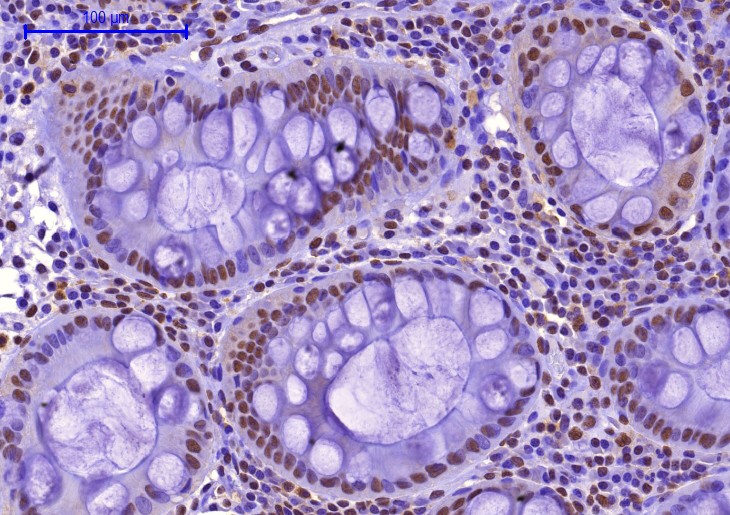


**A**


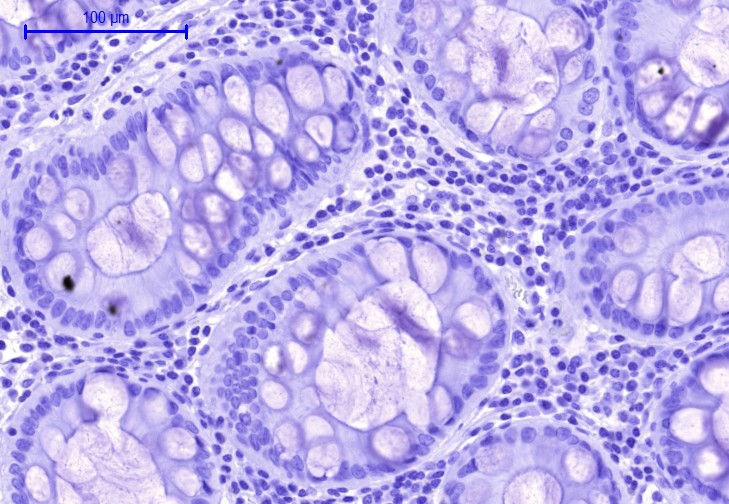


**B**

**S4 Fig.** Photomicrographs of positive (**A**) and negative (**B**) IHC staining with anti-MELK antibody in benign colon tissues as controls for the IHC detection of corresponding biomarker in breast cancer. (**A**) Antibody against MELK protein showed moderate nuclear staining in > 80% of benign colon crypts epithelium and lymphocytes. (**B**) For the negative control, isotype staining with corresponding immunoglobulin instead of antibody was used on adjacent slide. Original magnification x200.

| **S1 Table. Patient and tumor characteristics in association with *MELK* IHC expression** | | | | | | | | |
| --- | --- | --- | --- | --- | --- | --- | --- | --- |
| **Characteristic** | **Total,**  **n=46**  **n (%)** | ***MELK* nuclear expression** | | |  | ***MELK* cytoplasmic expression** | | |
|  |  | **Positive,**  **n = 39**  **n (%)** | **Negative,**  **n = 7**  **n (%)** | ***P*-value^2^** |  | **Positive,**  **n = 19**  **n (%)** | **Negative,**  **n = 27**  **n (%)** | ***P*-value^2^** |
| Age at Diagnosis |  |  |  |  |  |  |  |  |
| Median age (range), years | 54.5 (21-90) |  |  |  |  |  |  |  |
| ≤ 50 years old | 17 (37) | 15 (38.5) | 2 (28.6) | 1.000 |  | 6 (31.6) | 11 (40.7) | 0.555 |
| > 50 years old | 29 (63) | 24 (61.5) | 5 (71.4) |  |  | 13(68.4) | 16 (59.3) |  |
| Race |  |  |  |  |  |  |  |  |
| Black | 38 (82.6) | 32 (82.1) | 6 (85.7) | 1.000 |  | 15 (78.9) | 23 (85.2) | 0.839 |
| White | 6 (13) | 5 (12.8) | 1 (14.3) |  |  | 3 (15.8) | 3 (11.1) |  |
| Other | 2 (4.3) | 2 (5.1) | 0 (0.0) |  |  | 1 (5.3) | 1 (3.7) |  |
| Histologic Grade |  |  |  |  |  |  |  |  |
| Low | 4 (8.7) | 4 (10.3) | 0 (0) |  |  | 0 (0.0) | 4 (14.8) |  |
| Intermediate | 17 (37.0) | 15 (38.5) | 2 (28.6) | 0.828 |  | 7 (36.8) | 10 (37.0) | 0.316 |
| High | 22 (47.8) | 18 (46.2) | 4 (57.1) |  |  | 10 (52.7) | 12 (44.5) |  |
| Unknown | 3 (6.5) | 2 (5.0) | 1 (14.3) |  |  | 2 (10.5) | 1 (3.7) |  |
| Lymph nodes |  |  |  |  |  |  |  |  |
| Positive | 15 (32.6) | 12 (30.8) | 3 (42.9) |  |  | 6 (31.6) | 9 (33.3) |  |
| Negative | 31 (67.4) | 27 (69.2) | 4 (57.1) | 0.667 |  | 13 (68.4) | 18 (66.7) | 1.000 |
|  |  |  |  |  |  |  |  |  |
| Tumor size (cm) mean ± SD | 2.7± 1.9 |  |  |  |  |  |  |  |
| ≤ 2.0 cm | 20 (43.5) | 17 (43.6) | 3 (42.9) |  |  | 8 (42.1) | 12 (44.5) |  |
| > 2.0 cm | 25 (54.3) | 21 (53.8) | 4 (57.1) | 1.000 |  | 11 (57.9) | 14 (51.8) | 1.000 |
| Unknown | 1 (2.2) | 1 (2.6) |  |  |  | 0 (0.0) | 1 (3.7) |  |
| Pathologic stage |  |  |  |  |  |  |  |  |
| 0 | 3 (6.5) | 3 (7.7) | 0 (0.0) |  |  | 2 (10.5) | 1 (3.7) |  |
| I | 15 (32.5) | 13 (33.3) | 2 (28.6) | 0.912 |  | 5 (26.3) | 10 (37.0) | 0.750 |
| II | 17 (37.0) | 15 (38.5) | 2 (28.6) |  |  | 8 (42.1) | 9 (33.4) |  |
| III | 10 (21.8) | 8 (20.5) | 2 (28.6) |  |  | 4 (21.1) | 6 (22.2) |  |
| Unknown | 1 (2.2) | 0 (0.0) | 1 (14.2) |  |  | 0 (0.0) | 1 (3.7) |  |
| Estrogen Receptor (ER) Status |  |  |  |  |  |  |  |  |
| Positive | 26 (56.5) | 23 (59) | 3 (42.9) |  |  | 13 (68.4) | 13 (48.1) |  |
| Negative | 17 (37) | 14 (35.9) | 3 (42.9) | 0.667 |  | 5 (26.3) | 12 (44.5) | 0.219 |
| Unknown | 3 (6.5) | 2 (5.1) | 1 (14.3) |  |  | 1 (5.3) | 2 (7.4) |  |
| Progesterone Receptor (PR) |  |  |  |  |  |  |  |  |
| Positive | 24 (52.2) | 21 (53.8) | 3 (42.9) |  |  | 12 (63.1) | 12 (44.5) |  |
| Negative | 19 (41.3) | 16 (41.0) | 3 (42.9) | 1.000 |  | 6 (31.6) | 13 (48.1) | 0.351 |
| Unknown | 3 (6.5) | 2 (5.1) | 1 (14.3) |  |  | 1 (5.3) | 2 (7.4) |  |
| HER2 Status |  |  |  |  |  |  |  |  |
| Positive | 3 (6.5) | 1 (2.6) | 2 (28.6) |  |  | 1 (5.3) | 2 (7.4) |  |
| Negative | 40 (87) | 36 (92.3) | 4 (57.1) | 0.047 |  | 17 (89.4) | 23 (85.2) | 1.000 |
| Unknown | 3 (6.5) | 2 (5.2) | 1 (14.3) |  |  | 1 (5.3) | 2 (7.4) |  |
| Tumor subtype^1^ |  |  |  |  |  |  |  |  |
| Luminal A | 24 (52.2) | 22 (56.4) | 2 (28.6) |  |  | 12 (63.1) | 12 (44.5) |  |
| Luminal B | 2 (4.3) | 1 (2.6) | 1 (14.3) | 0.064 |  | 1 (5.3) | 1 (3.7) | 0.616 |
| HER2-positive | 1 (2.2) | 0 (0.0) | 1 (14.3) |  |  | 0 (0.0) | 1 (3.7) |  |
| Triple-negative (TNBC) | 16 (34.8) | 14 (35.9) | 2 (28.6) |  |  | 5 (26.3) | 11 (40.7) |  |
| Unknown | 3 (6.5) | 2 (5.1) | 1 (14.3) |  |  | 1 (5.3) | 2 (7.4) |  |
| ^1^Defined by 3-marker (ER/PR/HER2) IHC classifier  ^2^P-values are from Fisher’s exact test, excluding missing values | | | | | | | | |
